# UNDERSTANDING CAREGIVER PERSPECTIVES ON LIVE DISCHARGE AND RE-ENROLLMENT ONTO HOSPICE CARE

**DOI:** 10.1093/geroni/igac059.1128

**Published:** 2022-12-20

**Authors:** Stephanie P Wladkowski

**Affiliations:** Bowling Green State University, Bowling Green, Ohio, United States

## Abstract

Hospice care improves end-of-life outcomes for adults with Alzheimer’s Disease and related dementias (ADRD), yet with eligibility limited to a six-month prognosis, the patient-caregiver dyad can experience a live discharge from hospice. In 2019, nearly 350,000 patients with an ADRD diagnosis received hospice services in the US, and 6.5% of hospice patients were discharged due to being ‘no longer terminally ill.’ Caregivers of adults with ADRD who experienced a live discharge (n=24) were interviewed and thematic analysis was conducted. More than half (58%) noted specific support in their caregiving roles while 46% cited feeling relief. Eleven participants were enrolled for one hospice episode, and six re-enrolled at least one time. While participants would consider re-enrolling, they are waiting for a health crisis (n=7) for eligibility, while others question the meaning of hospice for ADRD patients (n=10). Implications for policy, practice, and research to support the patient-caregiver dyad are discussed.

